# Colistin Resistance Gene *mcr-8* in a High-Risk Sequence Type 15 Klebsiella pneumoniae Isolate from Kenya

**DOI:** 10.1128/MRA.00783-20

**Published:** 2020-09-24

**Authors:** Cecilia Kyany’a, Lillian Musila

**Affiliations:** a U.S. Army Medical Research Directorate-Africa, Nairobi, Kenya; Queens College

## Abstract

The emergence and rise of mobile colistin resistance genes are of great global concern due to the ease of transfer of resistance to other bacteria. This report describes the genome of a colistin- and multidrug-resistant Klebsiella pneumoniae isolate bearing *mcr-8*, obtained from a hospitalized patient in Kenya.

## ANNOUNCEMENT

Colistin is among the last-line drugs for the treatment of carbapenem-resistant bacterial infections. Mobile colistin resistance due to the *mcr* gene family is of global concern because the genes are easily transferred across species and strains. The *mcr-8* gene was first identified in Klebsiella pneumoniae isolates from humans in 2016 and from animals in 2018 in China (1). Since then, it has been reported in Asia (2) among sequence type 15 (ST15) (3) and ST39 (4) K. pneumoniae strains of human origin, Raoultella ornithinolytica (1), and Stenotrophomonas maltophilia (5). In Africa, *mcr* genes have been detected in South Africa (*mcr-1* and *mcr-9*) (6–8), Algeria (*mcr-8*) (9), and Tunisia (*mcr-1*) (10). Colistin is rarely used in Kenya; therefore, susceptibility testing is performed infrequently, and no *mcr* genes have been reported to date.

In April 2017, a 34-year-old male inpatient at a Nairobi hospital (1.2921S, 36.8219E) with a hip wound infection was enrolled in an antimicrobial resistance surveillance study. A swab sample from the infection site was cultured on blood agar and MacConkey agar. A single isolate was obtained, identified as K. pneumoniae, and tested against 17 antibiotics on a Vitek 2 system (bioMérieux, Marcy-l’Étoile, France) (Table 1). The isolate was nonsusceptible to drugs in five antibiotic classes (Table 1) and exhibited intermediate susceptibility to colistin on the Vitek 2 system (MIC, ≤0.5) and the Etest (bioMérieux) (MIC, >2) and resistance (MIC, >4) on the MicroScan system (Beckman Coulter, Brea, CA, USA) (11).

**TABLE 1:** Antibiotics tested and resistance genes identified in the K. pneumoniae isolate

Drug class	Drug(s) tested	Susceptibility test result^*a*^	Resistance gene(s)
Aminoglycosides	ND^*b*^	ND	*armA*, *aac(3)-IV*, *aac(6')-Ib-cr*, *aadA1*, *aadA16*, *aadA2*, *aph(3'')-Ib*, *aph(3')-Ia*, *aph(4)-Ia*, *aph(6)-Id*
Monobactams	Aztreonam	S	None identified
Carbapenems	Meropenem	S	None identified
β-Lactams (cephalosporins)	Ceftriaxone	S	*bla*_DHA-1_, *bla*_SHV-28_
	Cefepime	S	
	Cefuroxime	R	
	Cefixime	R	
Penicillins	Piperacillin	R	
	Ampicillin-sulbactam	R	
Fluoroquinolones	Levofloxacin	R	*oqxA*, *oqxB*, *qnrB2*, *qnrB4*
	Moxifloxacin	R	
Tetracyclines	Tetracycline	I	None identified
	Tigecycline	I	
	Minocycline	S	
Phenicols	Chloramphenicol	R	*cmlA1*, *floR*
Rifampin	ND	ND	*ARR-3*
Trimethoprim	Trimethoprim	R	*dfrA27*
Fosfomycin	ND	ND	*fosA*, *mdf*(A)
Macrolides	ND	ND	*mph*(E), *msrE*
Sulfonamides	ND	ND	*sul1*, *sul3*
Polymyxins	Colistin	S (I/R)^*c*^	*mcr-8*

a Resistance classifications are based on CLSI guidelines (16) and the Vitek 2 Advanced Expert System. S, susceptible; R, resistant; I, intermediate.

b ND, not done.

c The isolate tested intermediate on the Vitek 2 system and the Etest and resistant on the MicroScan system using the 2020 CLSI breakpoints (17).

DNA was extracted using the UltraClean microbial DNA isolation kit (MO BIO, Carlsbad, CA, USA); paired-end libraries were prepared using the KAPA HyperPlus kit (Roche, Basel, Switzerland) and sequenced using the MiSeq reagent kit v2 (300 cycles, 2 × 150 bp; Illumina, San Diego, CA, USA). Sequence reads were quality checked with FastQC v0.11.9, trimmed with Trimmomatic v0.39, and *de novo* assembled using SPAdes v3.13 (12) with default parameters. Multilocus sequence typing (MLST) was performed with the Institute Pasteur MLST database, and resistance genes and plasmid replicons were identified using ResFinder v3.2 (13) (Table 1) and PlasmidFinder v2.1 (14) (https://cge.cbs.dtu.dk/services/PlasmidFinder), respectively.

A total of 2,700,506 paired-end reads with a read length distribution of 139 to 151 bp were obtained. The total genome was 5.6 Mb, assembled into 105 contigs with an average GC content of 56.8% and coverage of approximately 130×. The isolate belonged to the hospital-associated high-risk ST15 group (15), and *mcr-8* was identified as the gene conferring colistin resistance. Plasmid replicons with a 100% match to IncR and IncHI1B, which were previously associated with *mcr-8*, suggested its genomic location on a plasmid.

This colistin-resistant isolate might have been missed based on the susceptible Vitek 2 MIC interpretation. A more sensitive colistin test for routine screening and caution when interpreting colistin results from existing automated platforms are recommended. This first description of an *mcr* gene in Kenya highlights the need to monitor emerging modes of colistin resistance and to limit the spread of high-risk clones.

The study was approved by the Kenya Medical Research Institute Scientific and Ethics Review Unit (protocol 2767), by the Walter Reed Army Institute of Research institutional review board (protocol 2089), and by the U.S. Army Medical Research and Materiel Command, Office of Research Protection, Human Research Protections Office (log A-18129). We have adhered to the policies for the protection of human subjects as prescribed in publication AR 70–25.

### Data availability.

This whole-genome shotgun project has been deposited at DDBJ/ENA/GenBank under the accession number JACHAY000000000. The version described in this paper is version JACHAY010000000. Raw sequence reads are available under the SRA accession number SRR11816972.
